# Managing the risk of cancer in Cowden syndrome: a case report

**DOI:** 10.1186/1752-1947-6-225

**Published:** 2012-07-30

**Authors:** Sonia Hammami, Olfa Berriche, Hichem Belhadj Ali, Olfa Hellara, Farooq Ansar, Silvia Mahjoub

**Affiliations:** 1 Department of Internal Medicine, University Hospital F. Bourguiba, Monastir, Tunisia; 2 Department of Dermatology, University Hospital F. Bourguiba, Monastir, Tunisia; 3 Department of Gastroenterology, University Hospital F. Bourguiba, Monastir, Tunisia; 4 Department of General Surgery, Warrington Hospital, Warrington and Halton Hospitals NHS Foundation Trust, Warrington, UK

## Abstract

**Introduction:**

Cowden syndrome is a rare cancer predisposition syndrome inherited in an autosomal-dominant fashion. The syndrome is characterized by hamartomatous polyps that affect multiple organs: skin, mucous membranes, thyroid, breast, gastrointestinal tract, endometrium and brain. It is also associated with an increased risk of developing malignancy in many tissues but especially breast, thyroid and endometrium.

**Case presentation:**

We present the case of a 30-year-old Tunisian woman with mental retardation who presented to our facility with rectal hamartomatous polyps. Her medical history included fibrocystic disease of the breast over the last three years. A physical examination revealed macrocephaly, hyperkeratotic papules on the mid-facial skin, palmoplantar keratosis and oral mucosal papillomatosis. A breast examination revealed nodular breast tissue bilaterally and a diffuse thyroid goiter. Our patient was clinically euthyroid. A total thyroidectomy was performed. A histopathologic examination revealed thyroid papillary carcinoma. A gastrointestinal evaluation revealed esophageal and gastric polyps. Biopsies showed hyperplastic and adenomatous lesions associated with *Helicobacter pylori*. A final diagnosis of Cowden syndrome was made according to the syndrome testing criteria adapted by the US National Comprehensive Cancer Network. A prophylactic bilateral mastectomy was proposed but refused by our patient. Our patient was kept under surveillance for breast and colorectal malignancies.

**Conclusions:**

Early and accurate diagnosis of Cowden syndrome is essential because it is a cancer predisposition syndrome that carries an increased risk for developing malignancy in many tissues, especially breast and thyroid. For this reason, education regarding the signs and symptoms of cancer is important. All patients must be screened for malignancies and options for prophylactic mastectomy should be discussed. Guidelines for cancer screening including surveillance and management plans for these patients should be distinguished from those of the general population, and may lead to a more timely diagnosis and treatment of cancers associated with this syndrome.

## Introduction

Cowden syndrome (CS) also known as multiple hamartoma syndrome, was first described in 1963 by Lloyd and Dennis referring to their patient Rachael Cowden, who died of breast carcinoma [1]. CS is a rare condition inherited in an autosomal dominant manner. The incidence is estimated to be one in 200,000 [2]. CS is characterized by mucocutaneous lesions, including facial trichilemmomas (hamartomas of the infundibulum of the hair follicle), oral papillomas and acral keratosis. Affected patients carry an increased risk of developing malignant lesions in many organs, but especially cancer of the breast, thyroid and genitourinary system [1]. We report a case of the disease, briefly review the current literature and discuss the management of the risk of cancer in CS.

## Case presentation

We present the case of a 30-year-old Tunisian woman with a history of multiple surgical procedures. She was born with congenital mental retardation. She had no significant family history except for renal nephrectomy in her brother when he was aged 27 years. She first presented to her medical practitioner at age 26 with altered bowel habits. An endoscopic examination at the time revealed rectal hamartomatous polyps treated with endoscopic polypectomy. The following year she presented with breast nodules on two separate occasions. Surgical excision was performed on each occasion and histology on each of these occasions revealed benign fibrocystic disease of the breast.

**Figure 1:**
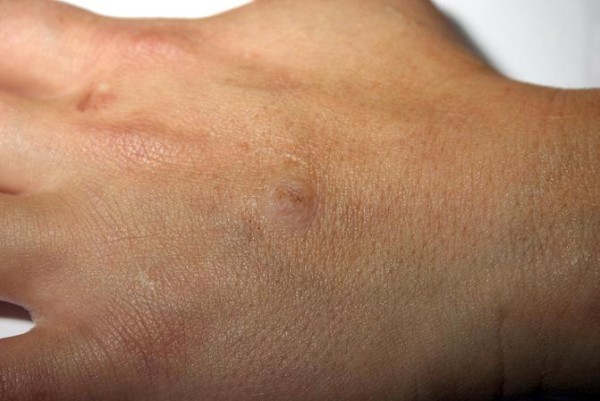
Our patient’s hands, showing keratosis.

During a routine follow-up examination at age 30 she was found to have multiple abnormal physical findings. She had craniofacial abnormalities with macrocephaly, a high-arched palate, adenoid facies, additional skin lesions with multiple facial keratotic papules, and papillomatosis, particularly around the eyes, mouth, and nostrils, buccal mucosal papules with a ‘cobblestone-like’ appearance, and cutaneous verrucous papules in the acral portions of the hands (Figure 1), feet, palms, and soles. A breast examination revealed multiple palpable nodules. There was clinical evidence of multinodular goiter, with a prominent nodule measuring 5.5cm in the left thyroid lobe. Our patient was clinically euthyroid. Additional physical findings included strabismus and skeletal abnormalities with kyphoscoliosis and pectus excavatum.

Laboratory investigations including renal biochemistry, liver function tests and thyroid hormones (T4, T3, and thyroid-stimulating hormone (TSH)) were normal. Results of a complete blood count showed iron-deficiency anemia.

**Figure 2:**
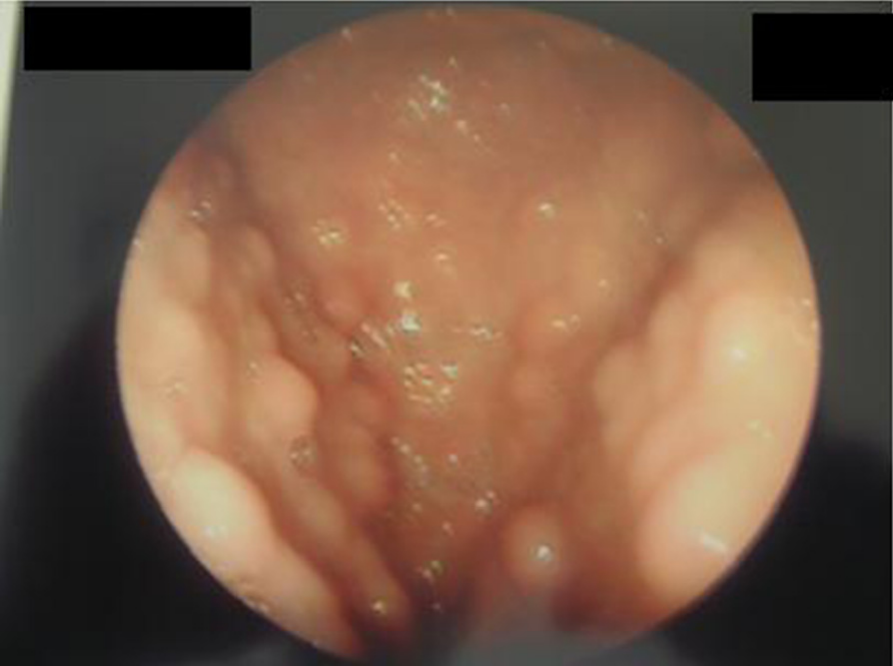
Endoscopic aspects of multiple esophageal polyps.

Excised buccal papules sent for histologic examination revealed acanthosis papillomatosis with keratosis epiderma. A breast ultrasound examination showed bilateral nodules compatible with fibrocystic disease. A total thyroidectomy was performed and histopathologic examination of the specimen revealed thyroid papillary carcinoma. Our patient received ablative therapy with iodine and was treated with levothyroxine. She has remained asymptomatic with undetectable serum thyroglobulin levels. A gastrointestinal evaluation with endoscopy revealed esophageal, duodenal, and gastric sessile polyps ranging from 1mm to 9mm in diameter. Biopsies showed hyperplastic and adenomatous lesions associated with *Helicobacter pylori* and esophageal lesions composed of glucogenic acanthosis. Colonoscopy diagnosed diffuse colorectal polyposis with multiple lesions in the sigmoid and rectum (Figure 2). Biopsies of these polyps showed inflammatory and hyperplastic hamartomatous polyps. Her iron-deficiency anemia was managed with iron supplementation. Our patient was diagnosed as having CS based on the syndrome testing criteria adapted by the US National Comprehensive Cancer Network (NCCN) [3]. A prophylactic bilateral mastectomy was proposed, but refused by our patient.

## Discussion

Cowden syndrome is an autosomal dominant syndrome predisposing to cancer, characterized by the occurrence of hyperplastic hamartomatous and tumoral lesions affecting various organs [2]. The disease mainly affects Caucasian women [1,4]. CS is most often diagnosed during the third decade of life (age range, 13 to 65 years) [1,4]. This syndrome is characterized by a combination of ectodermal, mesodermal, and endodermal alterations that may involve various organs: the skin, mucous membranes, breast, digestive tract, thyroid, and central nervous system. The characteristic skin signs such as facial trichilemmomas, acral keratosis and mucocutaneous papillomas, occur in 99% to 100% of patients and are preferentially localized in the peri-oral and facial regions [4]. These lesions are significant for diagnosis and have little malignant potential [5]. Breast lesions with fibrocystic disease, as observed in our patient, occur in approximately 75% of women. Breast carcinoma has been described in 30% to 50% of patients, and a recent review reported an 81% lifetime risk of breast cancer in patients with CS [6]. A literature search in PubMed revealed only six Tunisian patients with CS, three men and three women, ranging in age between six and 45 years. Investigation revealed thyroid lesions (three cases), but only one patient each had thyroid cancer, parotid carcinoma, and fibrocystic breast disease. No breast cancer was detected [7-9]. Environmental, genetic and nutritional factors may protect Tunisian patients with CS against breast cancer. Bennet *et al.* found that individuals with KILLIN-promoter methylation had a threefold increased prevalence of breast cancer over phosphatase and tension homologue on chromosome 10 (PTEN) mutation-positive individuals [10]. Prophylactic bilateral mastectomy may be recommended, but should be discussed on a case-by-case basis [2,4]. Surveillance consists of a monthly breast self-examination from age 18 years, biannual clinical breast examination starting at age 25 years or five to 10 years before the earliest age of diagnosis, annual mammography, and breast MRI screening starting at age 30 to 35 years or five years before the earliest age of diagnosis [3]. Recently, Riegert-Johnson *et al.* recommended breast screening at age 21 (nine years younger than recommended by the NCCN) [6].

Thyroid disease occurs in two-thirds of patients including goiter, thyroiditis, and thyroid cancer. Functional thyroid examinations should be performed with thyroid examination and ultrasound scan with biopsy of lesions recommended from age 10 years (eight years younger than recommended by NCCN) [3,6]. In case of anomalies, thyroidectomy is indicated [4].

Gastrointestinal involvement may be found throughout the gastrointestinal tract, frequently in the colon, but rarely in the small bowel [2]. Recent studies suggest that the gastrointestinal involvement is present at least 85% of patients in endoscopic screening [2,11]. Gastrointestinal involvement is predominantly in the form of hamartomatous colorectal polyps. Other polyps such as lipomatous, fibromatous, hyperplastic inflammatory and adenomatous lesions have also been described [11]. Esophageal glycogenic acanthosis is present in 40% to 60% of patients with CS and should be pathognomonic [11]. Until recently, it was reported that gastrointestinal involvement was not neoplastic. Heald *et al.*, in a prospective study of 127 PTEN mutation carriers reported that 13% of patients undergoing colonoscopy were diagnosed as having colorectal cancer [12]. This study confirms that patients with CS are at increased risk for cancer. Riegert-Johnson *et al.* reported cumulative cancer in a large group of patients with CS, confirming that patients are at increased risk for colorectal cancer (16%). According to the elevated colorectal cancer risk in CS, the authors recommended colonoscopy beginning at age 45 years and than every five years thereafter [6].

Additional findings include abnormalities of the female reproductive tract presenting as ovarian cysts (24%), leiomyoma (44%), and endometrial carcinoma (10%) [1]. Participation in a clinical trial is recommended for endometrial cancer screening to determine the effectiveness of screening modalities. Macrocephaly is present in approximately 80% of patients [1,4] and a high-arched palate in 15% and mild mental retardation is reported in 12% to 20% of cases [2]. In addition, central nervous system tumors, ganglioneuromas, neurofibromas, intra-cranial hypertension, granular cell myoblastoma and meningioma have all been reported in patients with CS [2]. Skeletal abnormalities are described in 37% of patients with CS including adenoid facies, kyphoscoliosis, syndactyly, and brachyphalangia. Other abnormalities include eye dysfunction, pulmonary lipoma, lung cysts, and cardiovascular problems [13].

Our patient had many of these documented physical findings of CS including skin and oral mucosal lesions, thyroid carcinoma, gastrointestinal polyps, skeletal abnormalities and a history of fibrocystic breast disease. A prophylactic bilateral mastectomy was proposed but refused by our patient.

Most patients with CS have a germ-line mutation in the tumor suppressor gene PTEN. The role of PTEN in tumorigenesis has been demonstrated and the loss of PTEN function contributes to cellular transformation, increasing the risk of cancer development in patients at an earlier age, such as in our patient, and with greater incidence than the general population. Patients are also at increased risk for premature death [4]. This mutation promotes cellular transformation and survival as well as resistance to chemotherapy and radiation. The mutation is identified in only 80% of patients who meet the clinical criteria. Tunisian CS cases suggest a PTEN dysfunction modulation that may protect against breast cancer. This hypothesis, based on only three women and three men with a short follow-up, should be interpreted with caution and needs to be confirmed.

The lack of genetic testing for our patient opens the possibility of other known genetic cancer susceptibility disorders. Thyroid carcinoma and colonic polyps and adenomas may be present in other genetic cancer predisposition disorders such as Muir-Torre syndrome; an autosomal dominant disorder characterized by sebaceous gland carcinoma and one or more internal visceral malignancies [14]. Newman *et al.* reported the case of a 52-year-old Caucasian women diagnosed as having sebaceous gland carcinoma of the eyelid, breast cancer and papillary carcinoma of the thyroid [15]. However, our patient fulfilled the criteria of CS adapted by the NCCN [3].

## Conclusions

We describe the case of a patient with CS initially presenting with fibrocystic breast disease and rectal hamartomatous polyps. Despite typical manifestations of CS, our patient was not correctly diagnosed initially. An early and accurate diagnosis of CS is essential to prevent cancers. Education and genetic testing of family members are recommended. The option of prophylactic mastectomy should be discussed in patients with CS; routine breast screening may be preferable.

## Consent

Written informed consent was obtained from the patient for publication of this manuscript and any accompanying images. A copy of the written consent is available for review by the Editor-in-Chief of this journal.

## Competing interests

The authors declare that they have no competing interests.

## Authors’ contributions

SH analyzed and interpreted the data from our patient and was a major contributor in writing the manuscript. OB analyzed and interpreted the data from our patient. HBA analyzed and interpreted the data from our patient. OH performed endoscopic explorations. AF contributed to the revision and writing of the manuscript. SM analyzed the data from our patient. All authors read and approved the final manuscript.
